# Normal interpupillary, inner canthal distance and outer canthal distance in a normal population of Pakistan

**DOI:** 10.12669/pjms.35.1.288

**Published:** 2019

**Authors:** Nausheen Hayat, Saba Alkhairy, Alyscia Cheema, Muneeb Ehsan, Muhammad Athar Khan

**Affiliations:** 1 Dr. Nausheen Hayat, FCPS, MRCSEd Opth, Consultant Ophthalmologist (Orbit Oculoplastic Surgeon), Jinnah Postgraduate Medical Centre, Karachi, Pakistan; 2 Dr. Saba Alkhairy, FCPS, Orbit Oculoplastic Surgeon, Assistant Professor, Ophthalmology Department, Dow University of Health Sciences, Karachi, Pakistan; 3 Prof. Alyscia Cheema, FCPS, FRCS Ed., Head of Department, Eye Ward. Jinnah Postgraduate Medical Centre, Karachi, Pakistan; 4 Dr. Muneeb Ehsan, MBBS, House Officer, Allama Iqbal Memorial Teaching Hospital, Sialkot, Pakistan; 5 Dr. Muhammad Athar Khan, MBBS, MCPS, DPH, DCPS-HCSM(MPH), MBA, PGD-Statistics, DCPS-HPE, Associate Professor, Department of Community Medicine Liaquat College of Medicine & Dentistry, Karachi, Pakistan

**Keywords:** Interpupillary distance (IPD), Inner canthal distance (ICD), Outer canthal distance (OCD)

## Abstract

**Objective:**

This study was conducted to quantify the normal indices of anthropometric measures related to ophthalmology including Interpupillary distance (IPD), Inner canthal distance (ICD), Outer canthal distance (OCD) in a normal, healthy Pakistani population.

**Methods:**

This is a cross sectional study. Total 500 patients were chosen randomly but 499 were included in this study. Patients were selected randomly in an outpatient department of Jinnah Post Graduate Medical Centre Karachi, over the period of five months. IPD, ICD & OCD all measurements were taken with the help of plastic rule by only one researcher to minimize chances of error as much as possible. IPD was reconfirmed from auto refractometer while ICD and OCD readings were taken twice by occluding one eye of researcher to reduce error. Participants were divided into four categories on basis of: Age, Gender, ethnicity and geographical location. Patients were further categorized on basis of Ethnicity to Urdu Speaking, Sindhi, Punjabi, Pathan, and others. Moreover, four age groups were drawn ranging from 15-24 years, 25-44 years, 45-64 years and 65 years and greater.

**Results:**

Our study comprised a total of 499 patents of which 272(54.5%) were males, and 227(45.5%) were females. The mean age of the participants was 39.3 ± 14.5 years. The mean values for the IPD, ICD and OCD in mm were 61.8 ± 6.2, 30.9 ± 2.9 and 85.2 ± 6.6 respectively. A statistically significant difference was observed between IPD, ICD and OCD Indices among male and female study participants (p<0.001, p=0.043, p<0.001). While comparing the IPD, ICD and OCD indices amongst the different ethnic groups, we found no statistically significant difference (p=0.09. p=0.28, p=0.06). Overall, there was no correlation between the age and other variables i.e. IPD, ICD, OCD, (r = 0.07, p = 0.085), (r = 0.005, p = 0.906), (r = -0.08, p = 0.058).

**Conclusion:**

This work has recommended normative values of IPD, ICD and OCD in Pakistani population on the basis different variables including gender, age, and ethnicity.

## INTRODUCTION

Anthropometry is a branch of anthropology concerned with measurements of the human body.^1^ There are certain anthropometric measurements in ophthalmology, required for identification of certain craniofacial syndromes and post traumatic abnormalities. Among these measurements normal IPD, ICD and OCD are the vital features to be known.

In order to have accurate assessment of telecanthus, hyper or hypotelorism we need to know standard values of IPD, ICD and OCD. Variations exist in these parameters as a result of difference in craniofacial growth due to racial, ethnic diversity, gender and age.^2^

By definition Interpupillary distance (IPD) can be defined as the distance between the center of the pupils.^3^ Far Interpupillary Distance is the IPD during measured when a person sees a distant object. In this study we measured far interpupillary distance. ICD is described as the distance between the medial canthi of both eyes. The OCD is the distance between the lateral canthi of the palpebral fissures bilaterally.^4^

To diagnose patients with hypertelorism, hypotelorism or telecanthus we require standard baseline values of above mentioned parameters in a normal Pakistani population. Previously, these conditions were assessed on the basis of clinical evaluation without any standard measurements, which creates a significant source of error in establishing a final diagnosis. As no work has been done related to this topic before in our department and institute we decided to design a study which can help us while working in our orbit oculoplastic clinic, to discrete abnormal patients from normal population.

We came up with objective to determine IPD, ICD and OCD in normal population of Pakistan and to compare these values by gender, age groups and ethnicity.

## METHODS

This cross sectional study was carried out in outpatient Department of Ophthalmology of largest tertiary care centre of Karachi, Jinnah Post Graduate Medical Centre (JPMC) over the period of five (5) months from April 2016 to August 2016. Approva lwas obtained from ethical committee of JPMC. Randomly 500 patients were targeted on the basis of following criteria, but 499 were selected. All Pakistani, male and female individuals between age of 15 years to 65 years and greater were eligible for inclusion. Transgender were excluded. Patients with neurological disease pertaining to eye, orbit or face, congenital or acquired craniofacial deformity, strabismus and orbital disease were excluded. All the patients were informed and consent has been taken before taking measurements. Plastic ruler was the main instrument used, but auto refractometer (RM 100 Topcon Company) was used for affirmation in few cases where readings were biased due to non-cooperative patients.

Each subject was seated comfortably in a chair with the subjects head at the same level as and 40 cm in front of the examiner’s head. The subject’s face was well illuminated. The examiner closed his one eye while asking patient to focus on a distant target and placed zero of plastic rule on forehead at temporal limbus of patient’s left eye and measuring the distance till nasal limbus of right eye, this measurement corresponds to the IPD. Readings taken were reconfirmed with auto refractometer but only plastic ruler measurements were included for data analysis.

Plastic ruler was placed on the root of the nose on forehead with its zero mark placing on inner canthus of right eye while examiner closing his right eye noted the distance between two inner canthi, labeling it as ICD. OCD was taken by placing plastic ruler at outer canthus of one eye and distance noted between two canthi while closing one eye of examiner.

It was found that while taking all measurements, closing one eye of examiner helped in sighting more precise readings rather than with both eyes open. All the above mentioned readings were taken twice by one examiner to reconfirm and avoid any kind of incongruity.

Data was obtained on the basis of three variable of age group, gender and ethnicity. Participants were further divided into four age groups 10-24 years, 25-44 years, 45-64 years and 65 years and greater. Ethnicity further categorized to Urdu Speaking, Sindhi, Punjabi, Pathan and others. Data were entered and analyzed using a Statistical Package for the Social Sciences (SPSS) software version 21. The normality of data was checked by Shapiro-Wilk test and result showed that the data was not normally distributed therefore non-parametric test was used for comparison. Categorical variables such as gender, ethnicity and age groups were presented as frequency and percentages while quantitative outcome variables IPD, ICD and OCD Indices as median, mean and standard deviation. Mann-Whitney Test was used to compare outcome variables with gender and Kruskal-Wallis Test for ethnicity and outcome variables while, Spearman’s correlation coefficient for age and IPD, ICD, OCD indices. P value of <0.05 was considered significant.

**Fig.1:**
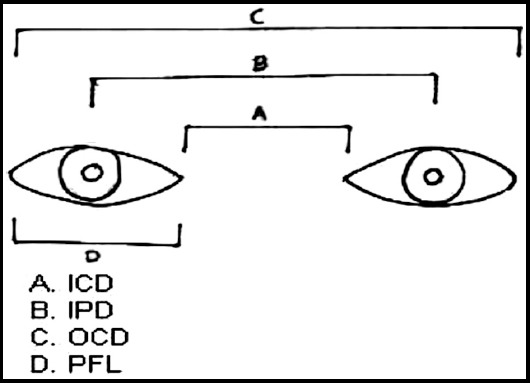
Anthropometric measurements of human eye showing inner canthal distance(ICD), interpupillary distance (IPD), outer canthal distance (OCD), palpebral fissure length(PFL).

## RESULTS

Our study comprised a total of 499 patents of which males 272 (54.5%) and females were 227 (45.5%). The mean age of the participants was 39.3 ± 14.5 years (Table-I).

**Table-I:** Demographic Characteristics of Study Participants (n=499), Descriptive analysis of demographic variables of participants for interpupillary, inner canthal and outer canthal distances.

Variable	N (%)
*Gender*	
Male	272(54.5)
Female	227(45.5)
*Ethnicity*	
Urdu Speaking	156 (31.3)
Sindhi	109 (21.8)
Punjabi	79 (15.8)
Pathan	74 (14.8)
Others	81(16.2)
*Age Groups(Years)*	
10-24 years	89 (17.8)
25-44 years	220 (44.1)
45-64 years	167 (33.5)
65 years & greater	23 (4.6)
Age in years (Mean ± SD)	39.3±14.5

The mean, median, maximum and minimum values for the IPD, ICD and OCD in mm are mentioned in Table-II.

**Table-II:** IPD, ICD and OCD Indices of Study Participants (n=499).

Variable	Mean ± SD	Median	Minimum	Maximum
Interpupillary Distance (mm)	61.8 ± 6.2	61	39	96
Inner Canthal Distance (mm)	30.9 ± 2.9	31	21	41
Outer Canthal Distance (mm)	85.2 ± 6.6	86	56	100

A statistically significant difference was observed between IPD, ICD and OCD Indices among male and female study participants as shown in Table-III.

**Table-III:** Comparison of IPD, ICD and OCD Indices among Male and Female study participants (n=499).

Variable		Mean ± sd	Median (min, max)	p-value*
Age of Participants	Male	38.6 ± 15.2	35 (15,72)	0.110
	Female	40.2 ± 13.6	40 (15,65)	
Interpupillary Distance (mm)	Male	62.7 ± 6.8	61 (39,96)	<0.001
	Female	60.7 ± 5.03	60 (51,88)	
Inner Canthal Distance (mm)	Male	31.2 ± 3.2	31 (21,41)	0.043
	Female	30.6 ±2.5	31 (23,39)	
Outer Canthal Distance (mm)	Male	86.3 ±7.0	87 (58,100)	<0.001
	Female	84 ± 6.0	85 (56,98)	

* Mann-Whitney U test.

While comparing the IPD, ICD and OCD indices amongst the different ethnic groups, we found no statistically significant difference as mentioned in Table-IV.

**Table-IV:** Comparison of IPD, ICD and OCD Indices among different ethnic groups (n=499).

		Mean ± sd	Median (min,max)	p-value*
Interpupillary Distance (mm)	Urdu Speaking	62.5 ± 6.7	61 (51,96)	0.09
	Sindhi	60.9 ±5.5	61 (39,90)	
	Punjabi	61.2 ± 6.5	60 (52,95)	
	Pathan	62.5 ± 6.6	61 (52,95)	
	Others	61.7 ± 4.8	61 (50,91)	
Inner Canthal Distance (mm)	Urdu Speaking	30.7 ± 3.0	31 (21,40)	0.28
	Sindhi	30.7 ± 2.9	31 (23,39)	
	Punjabi	30.8 ± 2.9	31 (21,39)	
	Pathan	31.4 ± 2.8	31 (24,40)	
	Others	31.0 ± 2.9	31 (24,41)	
Outer Canthal Distance (mm)	Urdu Speaking	85.3 ± 7.7	86 (56,99)	0.06
	Sindhi	84.8 ± 6.2	86 (60,94)	
	Punjabi	84 ± 6.1	85 (60,97)	
	Pathan	86 ± 6.1	86 (62,98)	
	Others	86.2 ± 5.7	86 (60,100)	

* Kruskal–Wallis test.

A spearman’s correlation coefficient was computed to assess the relationship between the age and IPD, ICD, OCD indices. Overall, there was no correlation between the age and other variables i.e. IPD, ICD, OCD, (r = 0.07, p = 0.085), (r = 0.005, p = 0.906), (r = -0.08, p = 0.058).

## DISCUSSION

It is essential to know standard Values of periorbital structure in different specialties such as occuloplasty and Orbital Surgery, optometry and genetics.^5^

A study in India has established that the mean values of ICD and OCD were 3.15 ± 0.2445 cm and 8.44 ± 0.3172cm respectively.^6^ These values were similar to the ones which we had calibrated during our study. Although the above study noted a difference in the values of ICD and OCD in males versus females which was consistent with our study. Another study done by Vasanthakumar P et al. showed the mean values of IPD, ICD and OCD to be 66.72 mm, 34.27 and 95.55 mm which were higher than ours.^7^ Another similar study in Pakistan showed the mean values of ICD, OCD and IPD were 3.4cm ±0.4cm, 10.7±3.9cm, 61.4cm±4.3cm among which only ICD values were similar to the values obtained in the Indian study.^8^

While comparing these parameters in males versus females we found a significant difference in the values of IPD, ICD, and OCD, p<0.001, p = 0.043, p<0.001 respectively. However the mean OCD value in males was 86.3±7.0 mm while in females it was less being 84± 6.0 mm. This result is agreeable to other studies like the one done by Evereklioglu C et al on the Turkish population which states that the mean OCD value was 87.07±4.36mm in males and 85.39±3.78mm in females.^9^ Siraj et al also confirmed this in his study on Sudanese population where the OCD in males were 92.23 mm and in female it was 90.79 mm.^10^

A study by Pakckiriswamy V on Malaysian population reported that the ICD in Malaysian South Indians was similar to Russians and Indians while the OCD was similar to Bulgarians, wider in Latvians and less than in Middle Eastern population.^11^ This was is accordance with a study done on the Nigerian population which reported a difference in the values of the mean canthal index amongst the ijaws and igbos.^12^

In our study we divided the participants into four age groups and measured these parameters to analyze the association of these measurements with age. The IPD was found to slightly increase from 61-63 mm in individuals 65 years or greater, while the ICD and OCD values remained the same with little variation along age. A Spearman’s correlation coefficient was computed to assess the relationship between the age and IPD, ICD, OCD indices. Overall, there was no correlation between the age and other variables i.e. IPD, ICD, OCD, (r = 0.07, p = 0.085), (r = 0.005, p = 0.906), (r = -0.08, p = 0.058). In a study done on Egyptian children aged between 1-13 years the IPD and OCD increased with age up till four years after which it started to decrease and then the ICD values remained the same up till 13 years of age.^13^ In a study done on Zairian children the ICD values were shown to progressively increase with age from 2 ^½^ years to 18 years.^14^ A study done by Muhammad Etezad reported a linear correlation between IPD and increasing age.^15^

The strength of our study over other similar studies was that we endeavored to analyze the difference in the measurement of IPD, OCD and ICD in different Ethnic groups which to our knowledge has not been done before in Pakistan. Although we suspected that there will be different values amongst the ethnic groups as the Balochi ethnicity people are broad boned and people from the Sindhi ethnicity are relatively small framed people yet we found no significant difference of these parameters.

### Limitation of study

It included small sample size and that it was a single center study. Further studies should encompass larger sample size and multicenter studies for more accurate results. We helped to provide normative values for the measured parameters which will serve as an important tool in diagnosing orbital dysmorphism as well as planning an oculoplastic or orbital surgery.

## CONCLUSION

This work has chalked out the normal values of IPD, ICD and OCD not only in gender but in different age groups and ethnicity of Pakistani population. This can help ophthalmologists and other specialty doctors in Pakistan understanding the features of normal population and lead them towards early diagnosis of hyper/ hypotelorism, telecanthus, beneficial in orbital surgeries and management of orbital and facial deformities. This manuscript can be valuable in forensic medicine and crime detection as it explains the features possessed by a large number of Pakistani people. Geneticists may take advantage in describing anomalies, human migration and evolution.
